# Identification of 14 Differentially-Expressed Metabolism-Related Genes as Potential Targets of Gastric Cancer by Integrated Proteomics and Transcriptomics

**DOI:** 10.3389/fcell.2022.816249

**Published:** 2022-02-21

**Authors:** Yongxin Zhang, Wenwei Liu, Wei Feng, Xiaofeng Wang, Tianxiang Lei, Zehong Chen, Wu Song

**Affiliations:** ^1^ Department of Gastrointestinal Surgery, The First Affiliated Hospital, Sun Yat-sen University, Guangzhou, China; ^2^ Laboratory of General Surgery, The First Affiliated Hospital, Sun Yat-sen University, Guangzhou, China; ^3^ Center for Digestive Disease, The Seventh Affiliated Hospital of Sun Yat-sen University, Shenzhen, China; ^4^ Department of Gastrointestinal Surgery, The Third Affiliated Hospital of Sun Yat-sen University, Guangzhou, China

**Keywords:** differential metabolic genes, proteomics, transcriptome, gastric cancer, BCAT2

## Abstract

Although research on the metabolism related to gastric cancer (GC) is gradually gaining increasing interest, there are few studies regarding metabolism-related genes in GC. Understanding the characteristic changes of metabolism-related genes at the transcriptional and protein levels in GC will help us to identify new biomarkers and novel therapeutic targets. We harvested six pairs of samples from GC patients and evaluated the differentially expressed proteins using mass spectrometry-based proteomics. RNA sequencing was conducted simultaneously to detect the corresponding expression of mRNAs, and bioinformatics analysis was used to reveal the correlation of significant differentially expressed genes. A total of 57 genes were observed to be dysregulated both in proteomics and transcriptomics. Bioinformatics analysis showed that these differentially expressed genes were significantly associated with regulating metabolic activity. Further, 14 metabolic genes were identified as potential targets for GC patients and were related to immune cell infiltration. Moreover, we found that dysregulation of branched-chain amino acid transaminase 2 (BCAT2), one of the 14 differentially expressed metabolism-related genes, was associated with the overall survival time in GC patients. We believe that this study provides comprehensive information to better understand the mechanism underlying the progression of GC metastasis and explores the potential therapeutic and prognostic metabolism-related targets for GC.

## Introduction

Gastric cancer (GC) is one of the leading causes of cancer-related deaths worldwide; however, the molecular mechanisms underlying GC remain largely unknown (Allemani et al., 2018). Although the combination of surgery and chemotherapy has shown great therapeutic progress, the prognosis of GC has still not significantly improved (Wei et al., 2020). The development of GC is a complex process in which a variety of molecules and signaling pathways are altered (Tan and Yeoh, 2015). Therapeutic strategies involving these molecules and signaling pathways could be promising for patients with GC. Therefore, it is necessary to elucidate the molecular mechanisms of GC to develop new biomarkers and therapeutic targets.

Many recent studies have proven that changes in metabolic pathways exist in many tumors and affect the malignant phenotype of tumors (Boroughs and DeBerardinis, 2015). The dysregulation of some metabolism-related genes has been observed in GC (Xiao and Zhou, 2017). As a key enzyme in the last step of glycolysis, pyruvate kinase M2 (PKM2) is highly expressed in GC, which can promote glycolysis and inhibit mitochondrial oxidative phosphorylation (Shiroki et al., 2017). PKM2 activates the PI3K/AKT pathway and inhibits autophagy, leading to the proliferative and invasive phenotype of GC cells (Wang et al., 2017). The upregulation of fatty acid synthase (FAS) in GC is closely related to lymph node metastasis (Ito et al., 2014). Therefore, this key enzyme related to lipogenesis has been studied as a potential target for anti-tumor therapy, and it is necessary to identify more differentially expressed metabolic genes in GC and normal gastric epithelial tissues as the molecular basis for targeted therapy.

In addition, the metabolites regulated by metabolic genes will have a profound impact on the function of immune cells in the tumor microenvironment (TME) (O’Sullivan et al., 2019). Tumor cells can deprive the TME of glucose through glycolysis to damage the function of immune cells such as T cells and NK cells (Ho et al., 2015; Cong et al., 2018). In addition, the massive consumption of some amino acids such as glutamine, serine or glycine or branched chain amino acids by tumor cells can impair the functions of T cells and NK cells, leading to immunosuppression environment (Ron-Harel et al., 2016; Swamy et al., 2016; Ma et al., 2017; Ren et al., 2017; Loftus et al., 2018). Hence, it is obvious that the competition for nutrients in the TME and the inhibitory effect of the metabolites on immune cells reshape the immune landscape. Understanding these processes will help us develop targets for tumor metabolites and improve the effectiveness of immunotherapy.

The latest advances in omics technology have led to a deep understanding of the molecular changes in the development and progression of cancer (Jiang et al., 2017; Dey et al., 2019). Therefore, multi-omics analysis in genomics, transcriptomics, epigenomics, proteomics, and metabolomics can help reveal key mechanisms in cancer development and treatment resistance to help guide treatment decisions. We believe that joint transcriptome and proteome profiling may reveal new biological insights and identify the pathogenic mechanisms or therapeutic targets for GC therapy.

## Materials and Methods

### Tissue Collection

This study was approved by the Research Ethics Committee of The First Affiliated Hospital of Sun Yat-sen University, and written consent was obtained from all patients. Six paired GC samples were used for proteomics analysis. Corresponding whole transcriptomics sequencing further confirmed the differentially expressed genes. All samples were collected from The First Affiliated Hospital of Sun Yat-sen University between January 2019 and December 2020. All tissues were histologically identified, diagnosed as gastric adenocarcinoma, and graded according to the guidelines of the modified American Joint Committee on Cancer (AJCC).

### Quantitative Proteomics by Multiplexed Tandem Mass Tag Mass Spectrometry

Proteins were extracted, digested with lysis buffer, and labelled with TMT reagents according to an optimized protocol (Pagala et al., 2015; Bai et al., 2017; Dey et al., 2019). The sample was fractionated using a C18 column (Waters BEH C18 4.6 × 250 mm, 5 μm) on a Rigol L3000 HPLC system. For transition library construction, shotgun proteomics analyses were performed using an EASY-nLCTM 1200 UHPLC system (Thermo Fisher, USA) coupled with a Q Exactive HF-X mass spectrometer (Thermo Fisher) operating in the data-dependent acquisition (DDA) mode. The identified protein contains at least one unique peptide with FDR no more than 1.0%. Proteins containing similar peptides that could not be distinguished by MS/MS analysis were identified as a same protein group. Reporter Quantification (TMT 10-plex) was used for TMT quantification. The protein quantitation results were statistically analyzed by Mann-Whitney Test, for proteins whose quantitation significantly different between GC and paired normal samples groups, (*p* < 0.05 and fold-change (FC) in expression ≥1.5), were defined as differentially expressed proteins (DEP). The detailed data processing procedure was presented in Supplementary Material S1.

### RNA-Seq

After total RNA was extracted, mRNA was isolated by Oligo Magnetic Beads and cut into small fragments for cDNA synthesis. Libraries were generated using the NEBNext UltraTM RNA Library Prep Kit (New England Biolabs, Ipswich, MA, USA) for the Illumina system following the manufacturer’s instructions. Sequencing was conducted using the Illumina HiSeq XTEN platform. The mRNAs with *p* < 0.05 between GC and paired normal samples were identified to be differentially regulated. The detailed data processing procedure was presented in Supplementary Material S2.

### Bioinformatics Analysis

The bioinformatics analysis was based on the online repositories including TCGA data portal (https://portal.gdc.cancer.gov/), TIMER database (https://cistrome.shinyapps.io/timer/), Kaplan-Meier Plotter database (http://www.kmplot.com/analysis/index.php?p=background) and GSCA database (http://bioinfo.life.hust.edu.cn/GSCA/#/immune).

### RNA Preparation and Reverse Transcription-Quantitative Real-Time PCR

All RNAs were isolated by RNA isolation plus (TaKaRa, Japan) according to the manufacturer’s protocol. cDNA was generated using PrimeScript RT Reagent (TaKaRa). The relative expression levels were measured by quantitative real-time reverse transcription polymerase chain reaction by using a LightCycler480 II Real-time PCR System (Roche, USA) with the SYBR green detection system (Takara). The samples were placed in a 96-well plate and amplified using the manufacturer’s standard amplification conditions (stage1:30 s at 95°C, stage2:40 cycles of 5 s at 95°C and 34 s at 60°C, stage3: Melt curve). Relative expression was determined by the 2^−ΔΔCT^ method. Meanwhile, we used GAPDH as an endogenous control for mRNA. The primer sequences used were as follows: BCAT2 (forward: 5′-GCC​CAC​CGT​GTT​AGT​GCA​A-3′, reverse: 5′-GTC​CAG​TAG​ACT​CTG​TCT​GAC​C-3′); GAPDH (forward: 5′CAA​GGT​CAT​CCA​TGA​CAA​CTT​TG-3′, reverse: 5′-GGC​CAT​CCA​CAG​TCT​TCT​GG-3′).

### Western Blot

For western blot analysis, total proteins were extracted using the Whole Cell Protein Extraction Kit (Key GEN, China). BCA Protein Quantitation Assay (Thermo, USA) was used to measure the protein concentration. We separated protein samples by using 10% sodium dodecyl sulfate-polyacrylamide gel electrophoresis (SDS-PAGE) gels. The separated protein samples were then transferred onto a polyvinylidene fluoride (PVDF) membrane (Millipore, USA). After blocking with 5% non-fat dry milk in Tris-buffered saline (TBS)/0.1% Tween 20 for 1 h at room temperature, the membranes were incubated with anti-BCAT2 (1:1,000, ab95976, Abcam, USA) and anti-GAPDH (1:1,000, 5,174, CST, USA) primary antibodies overnight at 4°C. The next day, membranes were washed 3 times with TBST buffer for 15 min and incubated with horseradish peroxidase-conjugated secondary antibody. Finally, the western blot signals were visualized using Immobilon Western Chemiluminescent HRP Substrate (Millipore, USA).

### Immunohistochemical Staining and Evaluation

The GC tissues were fixed in 4% paraformaldehyde and embedded in paraffin. The slides were then deparaffinized and heated in EDTA buffer for antigen retrieval. After being incubated with anti-BCAT2 (1:1,000, ab95976, Abcam, UK) at 4°C overnight, the slides were washed in PBS twice and subsequently incubated with HRP-conjugated secondary antibody (Abcam, UK) at room temperature. These samples were then visualized using diaminobenzidine (DAB), and the nucleus was stained with hematoxylin. The results of IHC were evaluated in a double-blind manner. We used semi-quantitative methods to determine staining scores, namely 0 (negative), 1 (weak), 2 (medium), and 3 (strong). Negative and weak staining confirmed low BCAT2 expression, while medium and strong staining indicated high BCAT2 expression.

### Cell Culture and Transfection

Gastric cancer cell lines, including AGS and HGC-27, were obtained from Procell Life Science & Technology Co., Ltd., Wuhan, China, Zhong Qiao Xin Zhou Biotechnology Co.,Ltd., Shanghai, China. All these cells were cultured in DMEM (Invitrogen, USA) supplemented with 10% fetal bovine serum (GBICO, USA) and incubated at 37 °C with 5% CO2.

### Plasmid Construction and Lentiviral Transduction

The plasmid pEZ-Lv201-CMV-BCAT2 was designed and synthesized by GeneCopoeia, Inc. a U.S. AGS and HGC-27 cells were transfected with Lipofectamine 2000 (Invitrogen, Carlsbad, CA, USA).

### Cell Counting Kit-8 Assay

The Cell Counting Kit-8 (CCK8, Dojindo, Japan) was used according to the manufacturer’s instructions. Briefly, 1,000 cells were seeded in a 96-well plate, and 10 μL of CCK-8 solution were added to each well every day. Wells were further incubated for 2 h and measured using an automatic microplate reader (Tecan Group Ltd., Männedorf, Switzerland).

### Colony Formation Assay

GC cell lines (AGS and HGC-27) resuspended to 1 ×  10^3^ cells/mL were seeded in 6-well plates. After incubation at 37 °C for 2  weeks, cells were fixed in 20% methanol for 30 min and stained with crystal violet for 20 min.

### 5-Ethynyl-2′-Deoxyuridine Assay

An EdU assay kit (RiboBio, Guangzhou, China) was to detect DNA synthesis and cell proliferation. Cells were seeded in a 96-well plate after 48 h of transfection and were continuously waited for 24 h. After incubation with 50 mM EdU for 2  h, the AGS and HGC-27 cells were fixed in 4% paraformaldehyde and stained with Apollo Dye Solution. Then, Hoechst 33342 was used to stain the nucleic acids. Images were obtained with a DMI8 microscope (Leica, Weztlar, Germany).

### Statistical Analysis

SPSS Statistics 20.0 (IBM, USA) and GraphPad Prism 6.0 (GraphPad Software, USA) were used for statistical analysis and graphing. The numerical data were presented as mean ± standard deviation (SD) of at least three experiments. Statistical comparisons between paired GC and normal gastric sample were performed using paired *t*-test comparisons. The Kaplan–Meier method was used to plot the survival curves, and the log-rank test was used to compare the differences between groups. *p* < 0.05 was considered to indicate statistical significance with a 95% confidence level.

## Result

### Headings

#### Proteomics Revealed a Special Metabolic Activity Characteristic in GC

**FIGURE 1:**
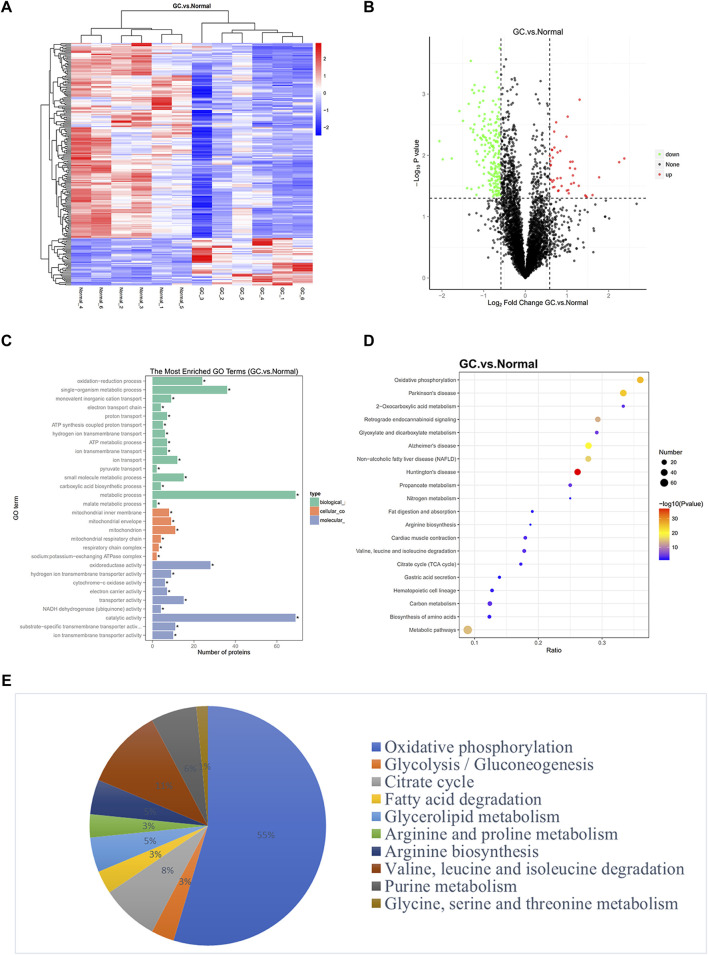
Identification of genes related to metabolic pathways through proteomics. **(A)** A cluster heat map presented the significantly dysregulated proteins in GC tissues related to paired normal tissues. The red and blue strips represented high and low expression, respectively. **(B)** Volcano plot filtering of differently expressed proteins between GC and normal gastric samples. High expression level is indicated by “red” and low levels by “green.” **(C–D)** GO pathway analysis and KEGG analysis showed the potential functions of differently expressed proteins in GC. The ratio in KEGG represents the protein ratio of pathways. **(E)** Enriched metabolic pathways exhibited distinct metabolic activities.

A variety of differentially expressed proteins can be detected in tissue samples as well as serum by TMT LC-MS/MS-based proteomics (Pagala et al., 2015; Dey et al., 2019). In this study, we screened differentially expressed proteins in GC using tandem mass tag–mass spectrometry (TMT-MS) analysis. The proteins with fold-change (FC) in expression ≥1.5 and *p* < 0.05 between GC and paired normal samples were identified to be differentially regulated. The 225 differently expressed proteins are listed in Supplementary Table S1 by FC. Hierarchical clustering and volcano plot filtering showed differently expressed proteins in GC (Figures 1A,B). Gene Ontology (GO) and Kyoto Encyclopedia of Genes and Genomes (KEGG) pathway analysis were conducted to evaluate the potential roles of these differentially expressed proteins (Figures 1C,D). Metabolic activity pathway was significantly enriched in these related pathways. Among these differentially expressed metabolic proteins, we observed a decrease in the level of mitochondrial oxidative phosphorylation (Figure 1E), which is consistent with previous studies on GC metabolism (Guaragnella et al., 2014). Considering that GC prefers glycolysis mode, known as “Warburg effect,” intervention in glycolysis metabolic mode of GC may be a promising therapeutic approach (Liberti and Locasale, 2016). In addition, the levels of lipids and triglycerides in GC generally rise due to inhibition of lipid degradation and enhanced lipid synthesis (Tugnoli et al., 2006; Leal et al., 2012). Our results also showed that the expression of PLPP2 and GK involved in the regulation of glycerolipid metabolism were upregulated, while the expression of ACAT1 and ECI2, involved in the fatty acid degradation pathway were downregulated. More importantly, our research revealed that amino acid metabolism proteins were widely dysregulated in GC, which was not the focus of previous studies on GC proteomics (Supplementary Table S2). Changes in arginine metabolism proteins were identified, in which ASS1 was significantly upregulated, while GPT2 and GLUL expression were downregulated. The high expression level of ASS1 in gastric cancer has been reported (Tsai et al., 2018), suggesting the important role of ASS1 in the metabolic process of gastric cancer. Studies have found that branched-chain amino acids (BCAAs) metabolic pathways are altered in many solid tumors such as melanoma, nasopharyngeal carcinoma, and breast cancer (Sivanand and Vander Heiden, 2020). Systemic metabolic disorders of BCAAs can affect the occurrence and progression of cancers such as pancreatic cancer (Falcone and Maddocks, 2020; Li et al., 2020), but it has not been reported in GC. In our study, proteins related to BCAA metabolism, including BCAT2, ALDH6A1, MCEE, PCCB, BCKDHB, DBT, and AUH, were all downregulated, revealing the potential role of BCAA metabolism in GC.

### Identification of Differentially Expressed Coupled mRNAs Using Transcriptomics in GC

**FIGURE 2:**
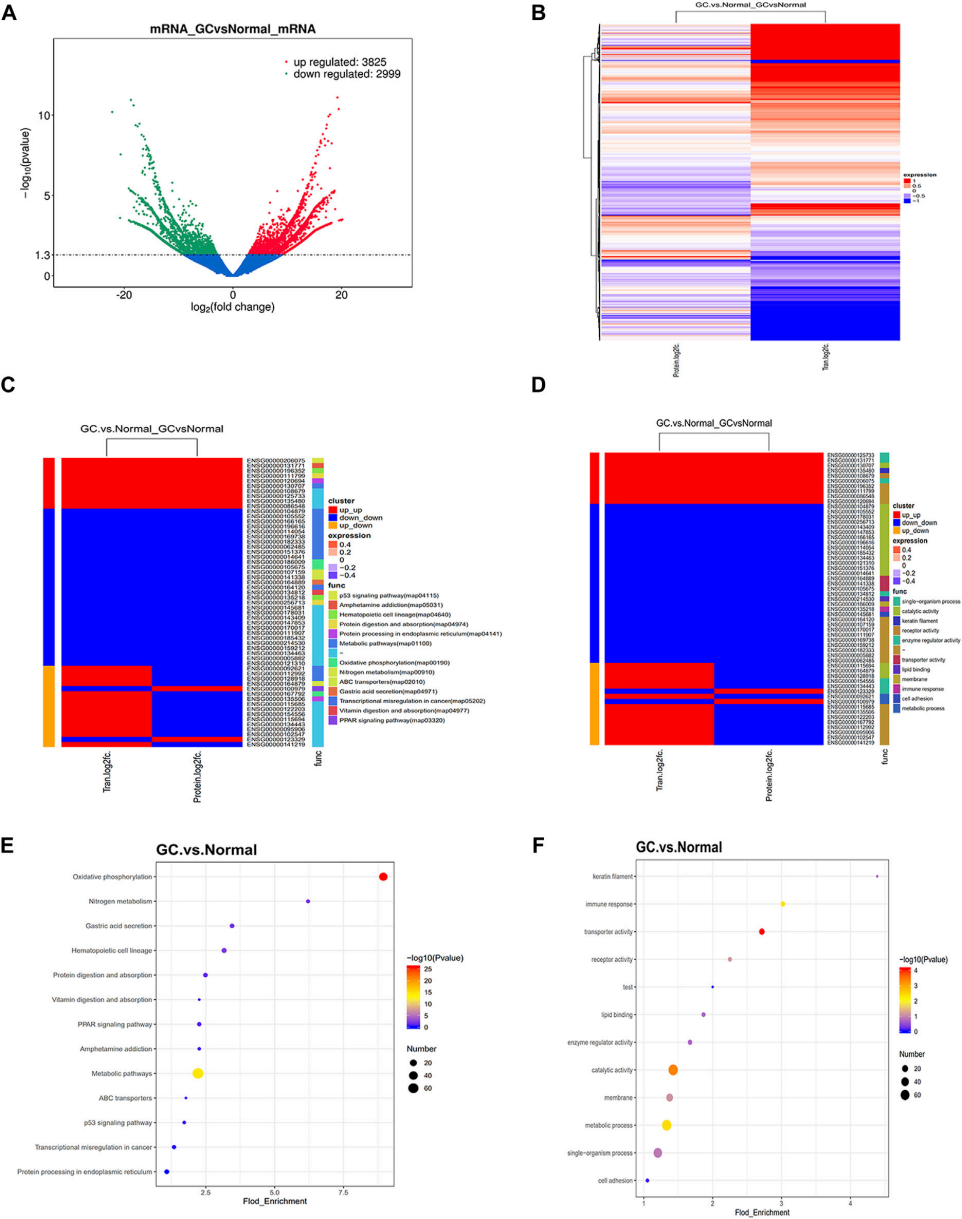
Identification of coupled genes through complementary transcriptomics. **(A)** Volcano plot filtering of differently expressed mRNAs between GC and normal gastric samples. High expression level is indicated by “red” and low levels by “green.” **(B)** Heatmap illustrating the different expression in protein and mRNAs between GC and normal gastric tissue. The red part represents up-regulated protein (mRNA) and the blue part represents downregulated protein (mRNA). **(C–D)** Clustering heat map of GO and KEGG pathway enrichment presents the potential functions of significantly dysregulated proteins (mRNAs) both in proteomics and transcriptomics. The red and blue strips represent high and low expression, respectively. **(E–F)** GO and KEGG pathway enrichment present the potential functions of significantly dysregulated proteins (mRNAs) both in proteomics and transcriptomics. The Fold_Enrichment represents the protein (mRNAs) ratio of pathways.

To complement the proteomic analyses, we performed RNA-seq (Figure 2A) and conducted correlation analysis of proteomics and transcriptomics (Supplementary Figure S1A). Heat map exhibited differentially expressed proteins (mRNAs) in proteomics and transcriptomics (Figure 2B). Finally, 57 significant differentially expressed proteins (mRNAs) were identified both in proteomics and transcriptomics (Supplementary Table S3; Supplementary Figure1B). We divided the 57 differentially expressed genes into three clusters. The mRNA and protein levels in cluster 1 and cluster 2 were coupled and the expression trend was the same, while the mRNA and protein levels in cluster 3 had the opposite trend. Next, we analyzed the potential function of 57 significant differentially expressed proteins (mRNAs). The clustering heat map of GO and KEGG pathway enrichment described the detailed pathway of every different expressed protein (mRNA) (Figures 2C,D). GO and KEGG pathway enrichment confirmed metabolic activity pathway was significant enriched (Figures 2E,F). Furthermore, KEGG pathway bias plot and scatter plot showed that the metabolic activity pathway was positively correlated in proteomics and transcriptomics (Supplementary Figures S1C, D). These 14 metabolic genes include *BCAT2*, *ALDH1A2*, *MDH1*, *PHGDH*, *CKB*, *ADH1B*, *PCCB*, *NNT*, *CKM*, *DCXR*, *LIPF*, *ASS1*, *ME3*, and *CS* that participate in various metabolic activities such as arginine, serine, branched chain amino acid, and tricarboxylic acid cycle, mostly focused on amino acid metabolism. As shown in Supplementary Figures S1E, the expression level of *ASS1* belonging to cluster 1 was upregulated, and the expression levels of *BCAT2*, *MDH1*, *CKB*, *ADH1B*, *PCCB*, *CKM*, *DCXR*, *LIPF*, *ME3*, and *CS* belonging to cluster 2 were downregulated at both the mRNA and protein levels. Genes belonging to cluster 3 such as *ALDH1A2*, *PHGDH* and *NNT* indicated that the mRNA level was upregulated and the protein level was downregulated, suggesting that these genes may be involved in post-transcriptional regulation.

### Bioinformatics Analysis of the 14 Metabolic Genes and the Relationship Between the Immune Cells

**FIGURE 3:**
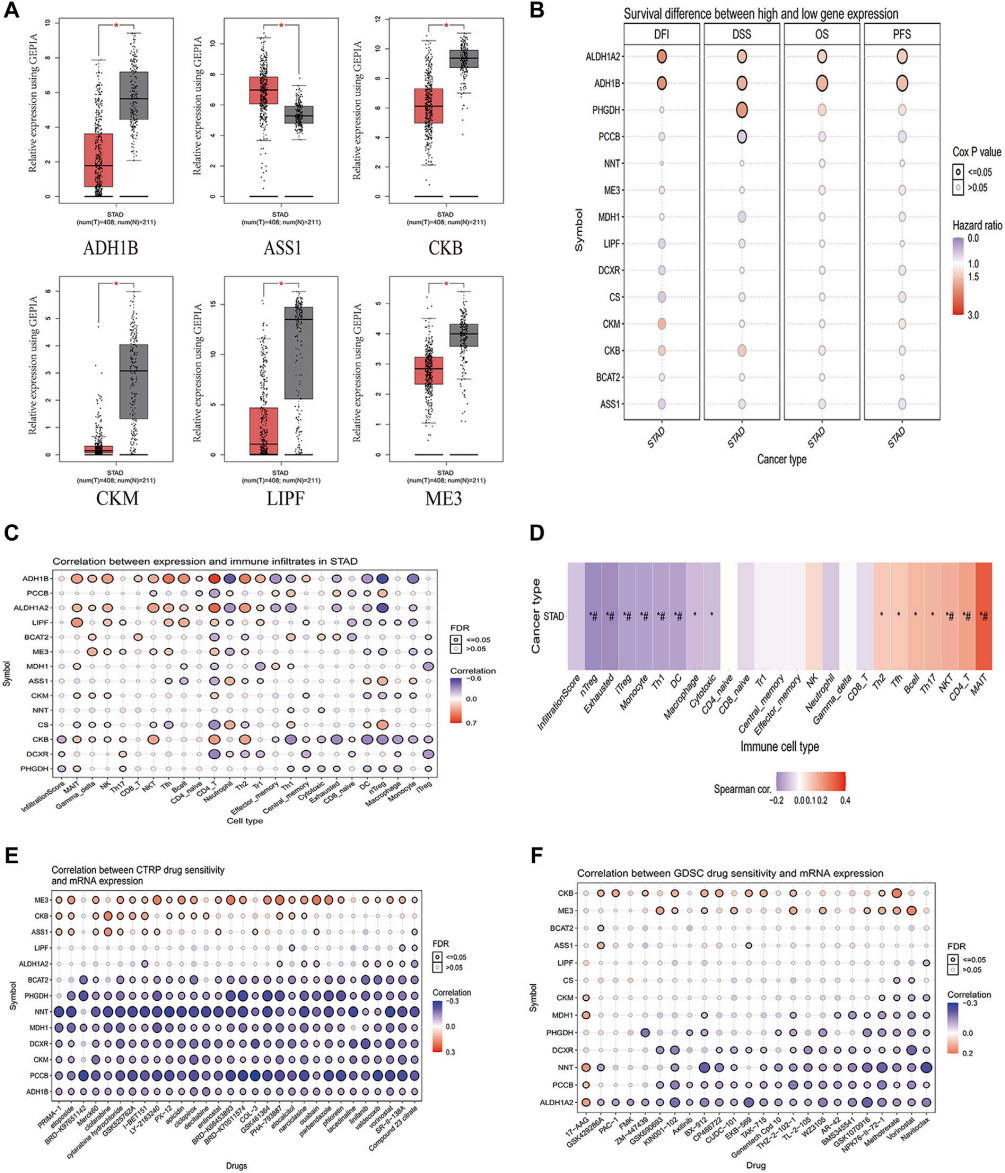
Analysis of the correlation between 14 metabolic genes and immunity. **(A)** Analysis of significantly different genes in unpaired GC (T = 408) and normal tissues (N = 211) in the GEPIA (*p* < 0.05). **(B)** Survival differential genes analysis in STAD from the GSCA database. **(C)** The correlation between 14 metabolic genes and immune cell infiltration in STAD from the GSCA database. **(D)** The correlation between a gene set composed of 14 metabolic genes and immune cell infiltration in STAD from the GSCA database. **(E)** Correlation analysis of 14 metabolic genes and CTRP drug sensitivity from the GSCA database. **(F)** Correlation analysis of 14 metabolic genes and GDSC drug sensitivity from the GSCA database. T, GC tissues; N, matched adjacent normal samples.

We used the TCGA database to analyze 14 metabolic genes, and the results showed that *ADH1B*, *CKB*, *CKM*, *LIPF*, and *ME3* were significantly downregulated and *ASS1* was significantly upregulated in GC (Figure 3A). Further survival analysis showed that *ADH1B*, *ALDH1A2*, and *PHGDH* were associated with poor Disease Free Survival (DSS), while *PCCB* was associated with better DSS (Figure 3B). Considering that tumors may deprive the microenvironment of nutrients *via* a variety of metabolic pathways and damage the function of immune cells, we analyzed the correlation between 14 metabolic genes and infiltrating immune cells in GC. The results showed that these metabolic genes are related to a variety of immune cells (Figure 3C). We grouped 14 metabolic genes into a gene set and found that the gene set was negatively correlated to Treg, monocytes, Th1, DC, and macrophages and positively correlated to NKT, CD4^+^, and MAIT cells (Figure 3D). Considering that these genes are generally downregulated in tumors, these genes may be associated with positive immune regulation. In addition, we evaluated the sensitivity of these metabolic genes to drug therapy, and the results showed that most genes are related to drug therapy sensitivity (Figures 3E,F), suggesting that targeting these genes can achieve anti-tumor effects through drug synergistic therapy.

### Correlation Analysis Between 14 Metabolic Genes and Programmed Cell Death Ligand 1/Cytotoxic T Lymphocyte-Associated Protein 4

**FIGURE 4:**
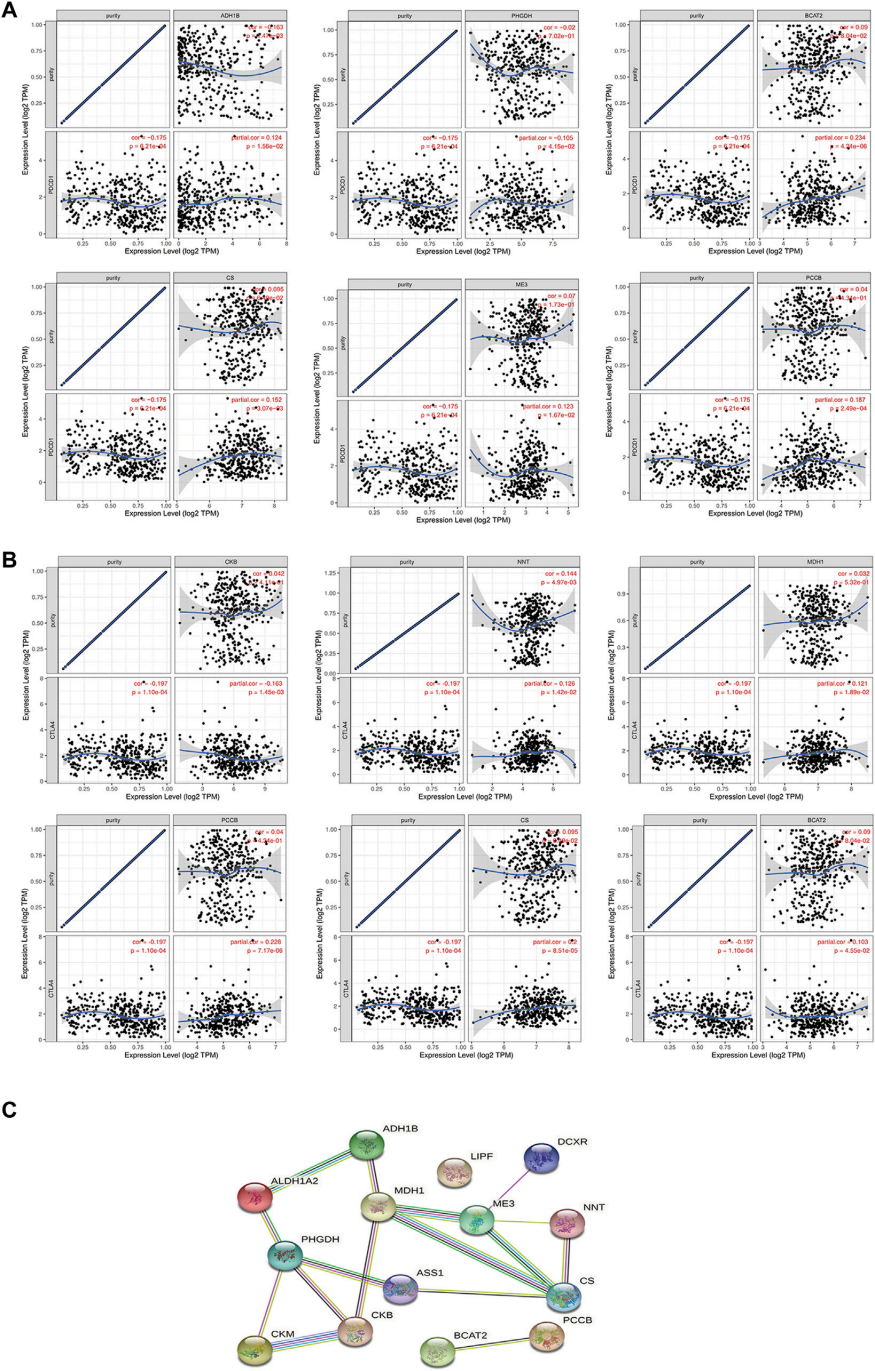
Analysis of the correlation between 14 metabolic genes and PD-1/CTLA4. **(A–B)** Correlation analysis of 14 metabolic genes and PD-1/CTLA4 in the TIMER database. **(C)** PPI network constructed by 14 metabolic genes in STRING.

Studies have shown that the metabolic interaction between tumor cells and immune cells may be related to poor response to immunotherapy. Therefore, targeting tumor metabolic activity including glucose or glutamine activity combined with PD-1/PD-L1 ICIs may provide new treatment opportunities for gastric cancer patients (Ma et al., 2021). We analyzed the co-expression relationship between 14 metabolic genes and PD-1/CTLA4 to help us understand whether these genes can be used as synergistic targets for immunotherapy. The results showed that *ADH1B*, *PHGDH*, *BCAT2*, *ME3*, *PCCB*, and *CS* were positively correlated with PD-1 (Figure 4A), and *ADH1B*, *PHGDH*, *BCAT2*, *CKB*, *PCCB*, and *CS* were positively correlated with CTLA4 (Figure 4B). Next, we constructed differentially expressed mRNA-mediated protein-protein interaction networks in GC to reveal their complex interactions among each other using the STRING system (Figure 4C).

### Identifying the Differential Expression and Prognostic Characteristics of BCAT2 in GC

**FIGURE 5:**
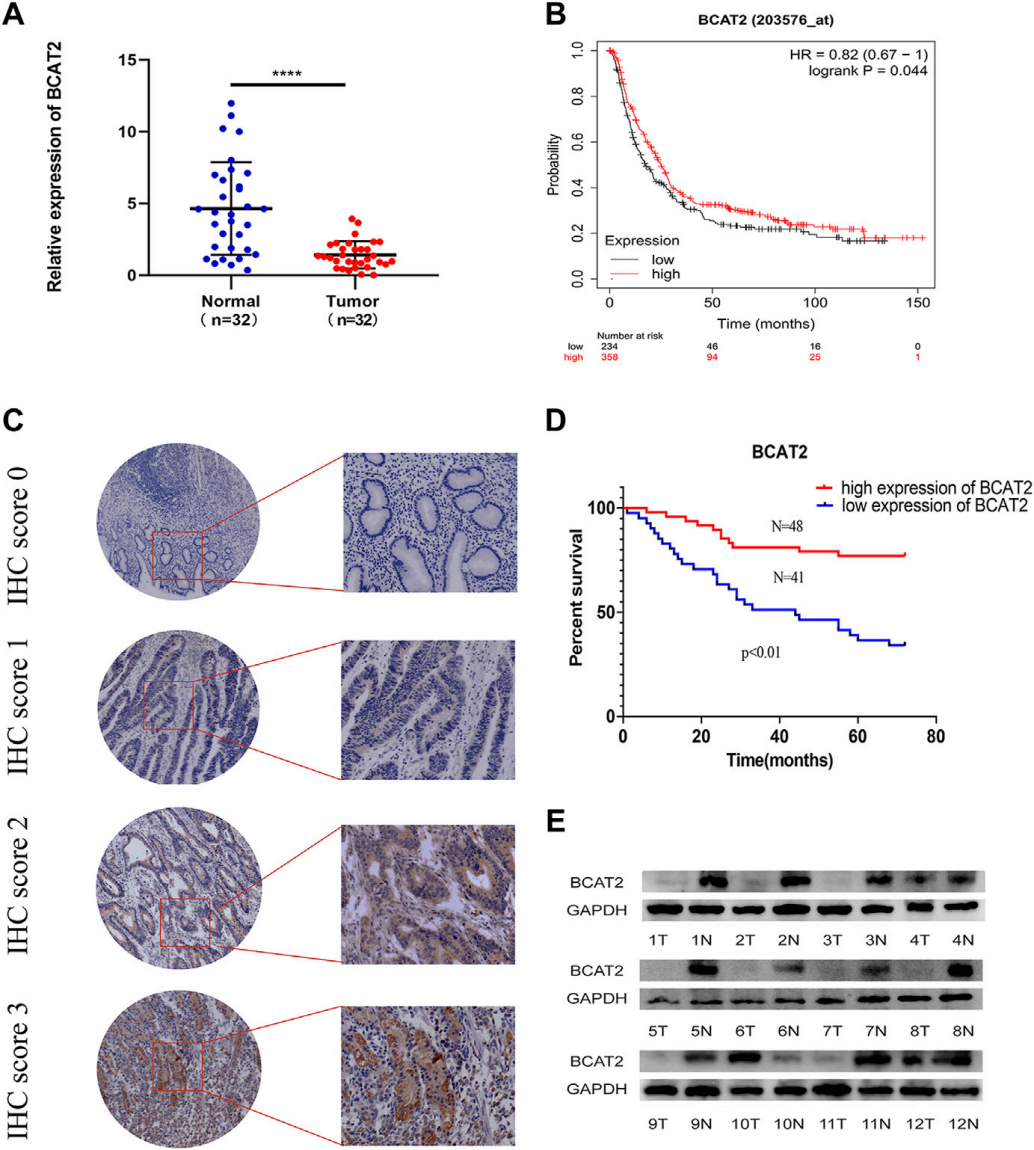
Low BCAT2 expression in GC. **(A)** Relative expression of BCAT2 in 32 paired GC tissues and matched adjacent normal samples *via* qRT-PCR. (*p* < 0.05). **(B)** The survival curve of BCAT2 in GC patients from the Kaplan–Meier plot (*p* < 0.05). **(C)** IHC staining of the BCAT2 protein in GC tissues. **(D)** The survival curve of BCAT2 in GC patients using the IHC staining score (*p* < 0.05). **(E)** The protein expression of BCAT2 in 12 paired GC tissues and matched normal adjacent mucosa, analyzed by western blotting. T, GC tissues; N, matched adjacent normal samples. *p < 0.05, **p < 0.01, ***p < 0.001, ****p < 0.0001.

**FIGURE 6:**
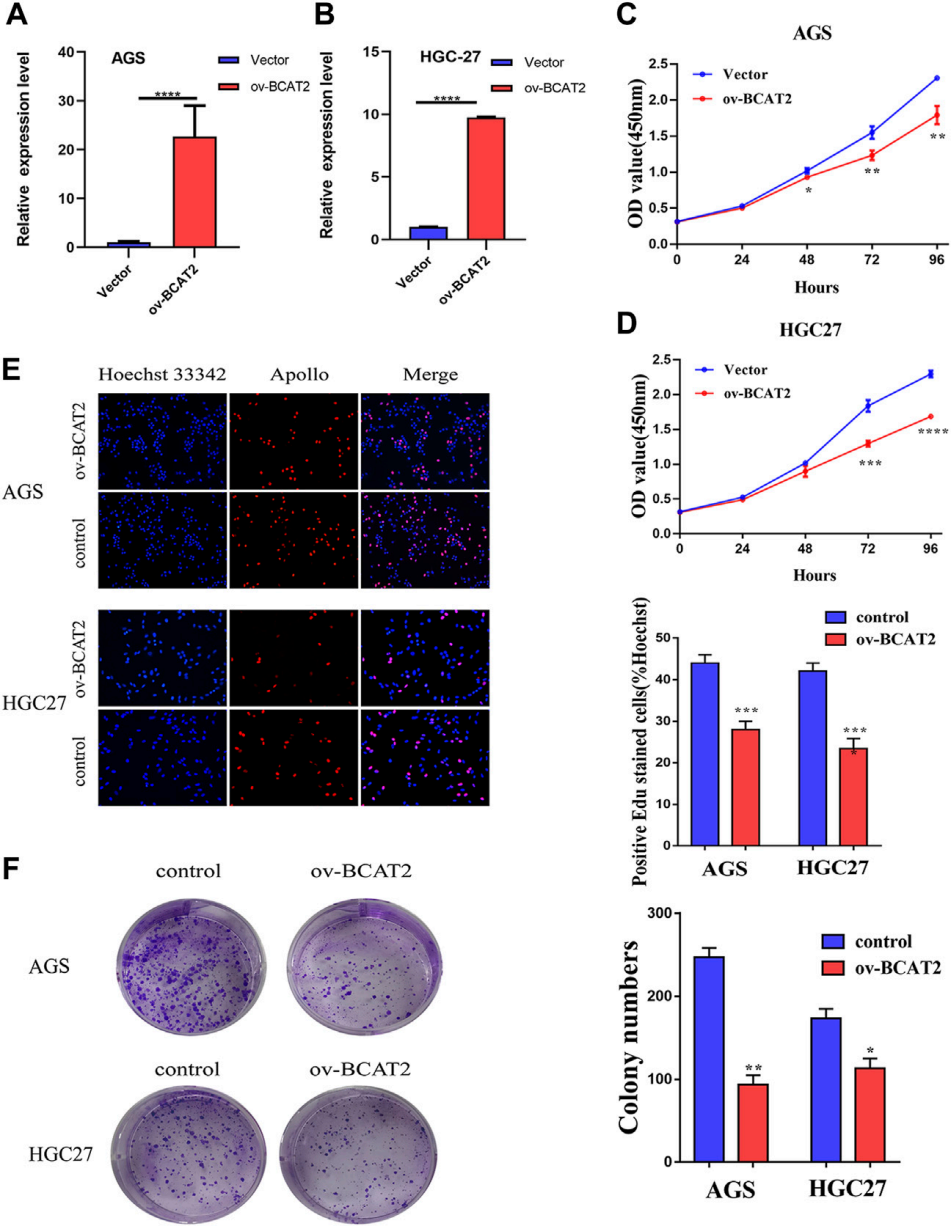
BCAT2 suppresses the proliferative ability of GC cells. **(A–B)** qRT-PCR analysis of BCAT2 mRNA expression after treatment with an overexpression plasmid in AGS and HGC-27 cells. **(C–D)** Assessment of the proliferation of AGS and HGC-27 cells transfected with control vector or BCAT2 plasmid by a CCK-8 assay. **(E–F)** Assessment of AGS cell proliferation by EdU and colony formation assays. Quantitative data from three independent experiments are shown as the mean ± SD (error bars). *p < 0.05, **p < 0.01, ***p < 0.001, ****p < 0.0001.

The mRNA level of BCAT2 were detected in 32 pairs of GC tissues and adjacent normal gastric mucosa by RT-qPCR (Figure 5A). BCAT2 was significantly decreased in GC compared to normal gastric epithelial tissue. By using the Kaplan-Meier Plotter database to compare the overall survival (OS) curve of BCAT2 expression (Figure 5B), the low level of BCAT2 is a signal of poor prognosis for GC patients. To further verify the difference in the expression of BCAT2 at the protein level, we performed western blotting on 12 pairs of matched GC and adjacent normal tissues (Figure 5E). We found that compared with adjacent normal gastric tissues, the levels of BCAT2 protein in GC tissues were significantly reduced. Thus, to explore the relationship between the expression of BCAT2 and the clinicopathological characteristics of GC, we performed IHC to detect the expression of BCAT2 in 89 GC tissues (Figure 5C). Negative and weak staining were classified as low BCAT2 expression (46.1%, 41/89), while moderate and strong staining were defined as high BCAT2 expression (53.9%, 48/89). As shown in Table1, low BCAT2 expression was associated with lymphatic invasion (*p* < 0.05). Further analysis showed that the 5-year overall survival rate of GC patients with high BCAT2 expression was significantly higher than that of GC patients with low BCAT2 expression (46.1% vs. 53.9%; *p* < 0.01, Figure 5D). To identify the functional effects of BCAT2 in gastric cancer cell lines, we transfected the BCAT2 overexpression plasmid into AGS and HGC-27 cells (Figures 6A,B). In the following steps, CCK8, Edu and colony formation assays were performed to determine the proliferative capacity of GC cells. We observed that the overexpression of BCAT2 inhibited cell proliferation rate, such as CCK8 assay (Figures 6C,D), DNA synthesis as measured by Edu assay (Figure 6E), and colony forming ability of AGS and HGC -27 (Figure 6F) cells. Therefore, our research shows that BCAT2 is a potential therapeutic target for GC.

**TABLE 1:** Relationships between BCAT2 expression and clinical-pathological parameters in gastric cancer.

Parameters	Group	*BCAT2* expression			
		Cases	Low	High	*p* value
Gender	Male	61	29	32	0.6806
	Female	28	12	16	
Age	<55	25	9	16	0.2337
	≥55	64	32	32	
Tumor size	≤4 cm	32	15	17	0.9088
	>4 cm	57	26	31	
Histology grade	Well-moderately	34	12	22	0.1089
	Poorly	55	29	26	
Tumor site	Cardiac	24	7	17	0.0519
	Non-cardiac	65	34	31	
Depth of invasion	T1+T2	16	4	12	0.0619
	T3+ T4	73	37	36	
Lymphatic invasion	N0	26	6	20	*0.0052
	N1-N3	63	35	28	
TNM stage	I + II	26	9	17	0.1638
	III + IV	63	32	31	

*p < 0.05.

## Discussion

The importance of metabolism in tumors is gradually being recognized, and metabolic activities are now understood to affect the malignant phenotype and immunosuppressive properties of tumors (Hanahan and Weinberg, 2011; Chang et al., 2015; Ho et al., 2015). Recently, Natalya N. Pavlova and Craig B. Thompson organized known cancer-associated metabolic changes into six hallmarks: 1) deregulated uptake of glucose and amino acids, 2) use of opportunistic modes of nutrient acquisition, 3) use of glycolysis/TCA cycle intermediates for biosynthesis and NADPH production, 4) increased demand for nitrogen, 5) alterations in metabolite-driven gene regulation, and 6) metabolic interactions with the microenvironment (Pavlova and Thompson, 2016). Compared to normal tissues, tumors exhibit enhanced nutrient absorption given the activation of oncogenes and the loss of tumor suppressor factors (Ying et al., 2012). However, nutritional limitations in solid tumors may require malignant cells to undergo metabolic reprogramming to provide sufficient energy and biosynthetic pathways (Gaglio et al., 2011; Son et al., 2013). In addition, the metabolic flexibility of tumor cells allows them to adapt to the diverse TME and achieve immunosuppressive effects by depriving glucose and by other methods to damage the functions of T cells, NK cells, macrophages, and DCs (Keating et al., 2016; O'Neill and Pearce, 2016; Badur and Metallo, 2018; Cong et al., 2018).

In our study, the results of combined proteomics and transcriptomics showed that genes related to metabolic pathways were significantly enriched, proving that metabolic genes play an important role in GC. The metabolic genes identified by our research are currently not comprehensive and specific to GC, and they can become promising targets for GC metabolism.

The metabolic regulation of GC includes four major categories—carbohydrates, amino acids, lipids, and nucleic acids—which are interconnected by intermediate products (Xiao and Zhou, 2017). GC exhibits the Warburg effect, which involves high glucose uptake, enhanced glycolysis, and accumulation of large amounts of lactic acid. Tumor-derived lactic acid impairs the function of cytotoxic T cells/NK cells (Fischer et al., 2007) and prevents the differentiation of monocytes into DCs (Gottfried et al., 2006), ultimately leading to tumor immune escape. In addition to glycolysis, the effects of amino acid metabolic reprogramming on oncogenesis and immune evasion in GC have been gradually revealed. The kynurenine pathway catalyzed by indoleamine-2, 3-dioxygenase (IDO) plays a key role in regulating the TME to promote cancer progression. Higher expression of IDO is associated with increased activity of immunosuppressive T regulatory cells (Bauer et al., 2005). Regulating amino acid levels in TME may be an effective way for tumors to regulate immune cell function. For example, tumor expression of tryptophan depleting enzyme IDO and subsequent production of kynurenine can lead to the inhibition of T-cell proliferation and effector function and damage to DCs.

Using proteomics and transcriptomics, we identified 14 metabolic genes that are involved in multiple metabolic pathways. As the six hallmarks of tumors mentioned above, PHGDH was proven to be involved in use of glycolysis/TCA cycle intermediates for biosynthesis and NADPH production, and ASS1 was proven to be involved in increased demand for nitrogen (Pavlova and Thompson, 2016). We also demonstrated that these metabolic genes are related to a variety of immune cells. A gene set including 14 metabolic genes was negatively related to Treg, monocytes, Th1, DCs, and macrophages, and was positively correlated to NKT, CD4^+^, and MAIT cells. For example, PHGDH supports the rapid growth and uncontrolled spread of a variety of cancers by catalyzing the first step reaction of serine biosynthesis (Zhao et al., 2021). However, serine is also a key immune metabolite that directly regulates immune activity by controlling the proliferation of T cells. The lack of serine in the TME caused by the upregulation of PHGDH can impair the function of immune T cells. The expression of PHGDH was negatively correlated with the 5-year survival rate of GC patients, and multivariate analysis shows that it was an independent prognostic factor for the prognosis of GC (Xian et al., 2016). However, the effect of high expression of PHGDH on the GC TME and immune cells has not yet been studied. In addition, ASS1 plays a dual role in tumor cells. ASS1-low tumor cells become very dependent on external arginine, forming the basis of arginine deprivation therapy (Jahani et al., 2018). In ASS1-high tumor cells, such as prostate cancer (Gannon et al., 2010), breast cancer (Cavdar et al., 2003) and renal cell carcinoma (Tate et al., 2008), the presence of arginase in the TME can cause adverse effects, especially regarding the immune response to cancer cells. ASS1-expressing tumors recruit certain cells such as tumor-associated macrophages (TAM) and bone marrow-derived suppressor cells (BMDSCs), which can promote immune evasion (Adams et al., 2015). ASS1 is related to the production of polyamines and inhibition of NO production by macrophages involved in inflammation, which ultimately leads to the consumption of arginine in the TME (Chang et al., 2001). It is well known that arginine can enhance the immune response by promoting the survival and proliferation of T cells, and arginine deprivation may lead to immunosuppression (Cao et al., 2016; Geiger et al., 2016). ASS1 was highly expressed in GC and could promote invasion and metastasis, which proved that GC has unique metabolic characteristics (Tsai et al., 2018). However, the specific mechanism of ASS1’s role in GC microenvironment needs further exploration. We believe that the 14 identified metabolic genes by multiomics will help strengthen the understanding of GC.

We also found that some metabolic genes such as *ADH1B*, *PHGDH*, *BCAT2*, *ME3*, *PCCB*, and *CS* were positively correlated with PD-1. PD-1 inhibitors such as pembrolizumab have been used as a third-line drug for the treatment of GC, revealing the important role of immunotherapy in GC (Zhu and Ma, 2021). Research has shown that the combined use of PD-1 with small molecule drugs targeting metabolic pathways including amino acid metabolism may contribute to the effectiveness of PD-1 therapy. For patients with PD1 targeted drugs showing sustained response to treatment, the tumor mainly has a T cell-inflamed TME (Lanitis et al., 2017). The metabolic-related pathway such as tryptophan-kynurenine-arene receptor (Trp-Kyn-AhR) in T cell-inflamed tumors mediates a variety of immunosuppressive mechanisms including the consumption of tryptophan, direct immunosuppression of Kyn, and the activity of AhR bound to Kyn (Labadie et al., 2019). Small molecule inhibitors of this pathway are making progress in preclinical development and are expected to be used in combination with PD-1 checkpoint inhibitors to enhance the effect of PD-1. Therefore, these metabolic pathway genes including *ADH1B*, *PHGDH*, *BCAT2*, *ME3*, *PCCB*, and *CS* may become effective targets for synergistic therapy with PD-1.

In this study, based on the results of proteomics and transcriptomics, we found that BCAT2 was downregulated in tissues from GC patients and was significantly associated with a poor prognosis. As a type of branched-chain amino acid transferase, BCAT2 reversibly converts branched-chain amino acids into the corresponding branched-chain α-keto acid to generate glutamate (Neinast et al., 2019). Recently, BCAA metabolism has attracted widespread attention. The way different tumors utilize BCAAs exhibits tissue-of-origin dependence. Despite that, KRAS and TP53 mutations are both important genetic events in non-small cell lung cancer (NSCLC) and pancreatic ductal adenocarcinoma (PDAC); NSCLC tumors exhibited enhanced BCAA uptake, while PDAC tumors showed decreased BCAA uptake (Mayers et al., 2016). However, they further found that PDAC BCAT null cells formed smaller tumors in the pancreas than control cells, demonstrating that the growth of PDAC tumors may be aided by BCAT activity in certain tissue environments. As a solid tumor, the complicated TME of GC affects the changes of metabolic pathways. Therefore, it is necessary to understand the metabolism of BCAA *via* BCAT2 in different contexts in GC.

A study by Li et al. also confirmed that BCAT2 promoted the growth of pancreatic tumors by mediating BCAA catabolism and mitochondrial respiration (Li et al., 2020). I In addition, Wang et al. found that sorafenib and sulfasalazine could downregulate the expression of BCAT2 to induce iron death, thus identifying BCAT2 as a novel inhibitor of iron death (Wang et al., 2021). BCAT2 plays different roles in a variety of tumors, and its specific mechanism of action in GC has not yet been elucidated. Changes in metabolic genes and products can be used to promote the malignant phenotype and proliferation of tumors. Recent studies have shown that leucine is an activator of the mTOR pathway (Wolfson et al., 2016), indicating that GC may reduce the expression of BCAT2 to accumulate more leucine and activate mTOR to promote the growth of GC.

In conclusion, our research provided insights that reveal the characteristics of genetic alterations in GC metabolism. Despite the lack of large-scale sample verification and consideration of intra-tumor and inter-tumor differences, we believe that metabolic genes play an important role in GC adaptation to TME and immune resistance, which will be verified in more large-scale studies in the future.

## Data Availability

The datasets presented in this study can be found in online repositories. The names of the repository/repositories and accession number(s) can be found below: Gene Expression Omnibus, GSE193453.

## Supplementary Material

Supplementary Figure S1
**(A)** Plot of correlation analysis of proteomics and transcriptomics between GC and normal gastric samples. The green point represents a protein (mRNA) with significant difference in expression, and the blue point represents a protein (mRNA) without significant difference in expression. Log 2 (FC) was used for calculation of protein (mRNA) expression. **(B)** Venn plot of the transcriptome and proteome between the GC and normal gastric samples. all_Tran represents all genes from the transcriptome, diff_Tran represents differentially expressed mRNAs identified by the transcriptome, all_Prot represents all proteins identified by the proteome, and diff_Prot represents the differentially expressed protein identified by the proteome. Fifty-seven differentially expressed proteins (mRNAs) were identified both in the proteomics and transcriptomics. **(C)** The KEGG pathway bias plot shows the detailed pathway of dysregulated proteins (mRNAs) both in proteomics and transcriptomics. The biased-ratio represents the ratio of dysregulated proteins (mRNAs) with positive/negative correlation in the total dysregulated proteins (mRNAs). **(D)** The scatter plot shows the metabolic activity pathway exhibited a positive correlation in proteomics and transcriptomics. **(E)** Heat map of 14 metabolic gene expression in proteomics and transcriptomics.
