# Supplementary figures and images for: Identification of 14 Differentially-Expressed Metabolism-Related Genes as Potential Targets of Gastric Cancer by Integrated Proteomics and Transcriptomics
Source: Front Cell Dev Biol. 2022 Feb 21;10:816249. doi: 10.3389/fcell.2022.816249 (PMC8899292; doi:10.3389/fcell.2022.816249)

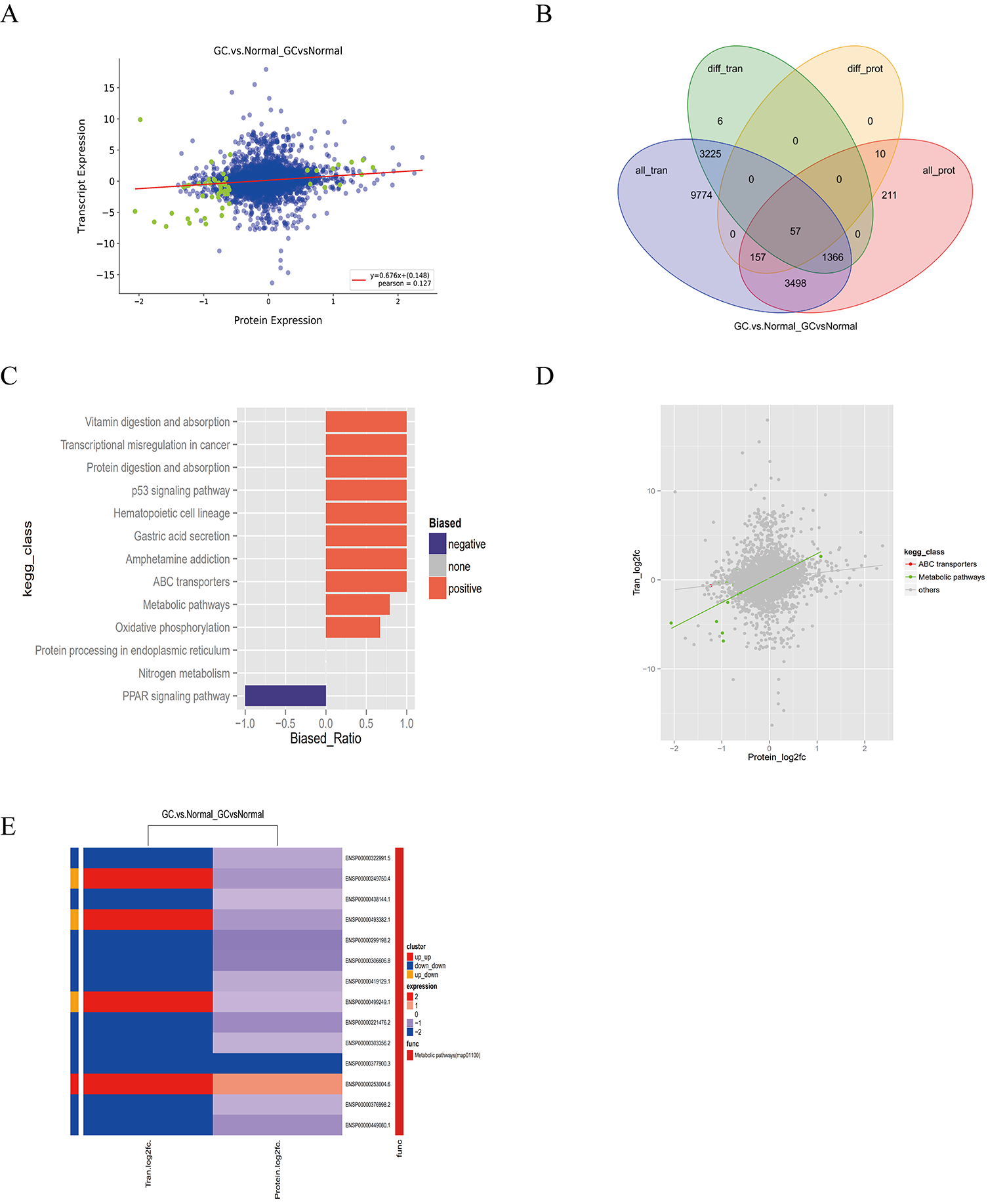

Supplement: Supplementary file 1 [file Image1.TIF]
